# Ipecac as a Hæmostatic

**Published:** 1879-12

**Authors:** 


Ipecac as a Hæmostatic.—Pecholier (*Jour. des Sci. Med.*, 1879, p. 513; from *Bull. Gen de Therap.*) speaks of the singular property possessed by ipecac of chasing, so to speak, the blood from the lungs. That is not due to the vomitive action is probable from the fact that tartar emetic does not seem to act in the same manner. The pulmonary anæmia is due, according to Pecholier, to a special action of the ipecac quite distinct from its nauseating or vomitive properties. The dose given (ʒi) is not, strictly speaking, a vomitive dose, and even when vomiting is produced, a certain amount of drug enters the general circulation. Pecholier's favorite prescription is as follows:

℞—Ipecacuanhæ contus....................ʒiss

Aquæ bulliente..........................℥ix

Make an infusion; filter; add syrupi acaciæ f℥i. Give a tablespoonful every hour or two.

The first dose may cause vomiting, but this soon ceases, and, indeed, may be prevented by the addition of a few drops of laudanum. The absorption of the emetic being thus rendered easier, pulmonary anæmia is rapidly and surely produced.—*Philad. Med. Times.*

